# An Embedded Sensor Node Microcontroller with Crypto-Processors

**DOI:** 10.3390/s16050607

**Published:** 2016-04-27

**Authors:** Goran Panić, Oliver Stecklina, Zoran Stamenković

**Affiliations:** IHP, Im Technologiepark 25, 15236 Frankfurt, Germany; stecklina@ihp-microelectronics.com (O.S.); stamenko@ihp-microelectronics.com (Z.S.)

**Keywords:** sensor node, security, cryptography, processor, system-on-chip

## Abstract

Wireless sensor network applications range from industrial automation and control, agricultural and environmental protection, to surveillance and medicine. In most applications, data are highly sensitive and must be protected from any type of attack and abuse. Security challenges in wireless sensor networks are mainly defined by the power and computing resources of sensor devices, memory size, quality of radio channels and susceptibility to physical capture. In this article, an embedded sensor node microcontroller designed to support sensor network applications with severe security demands is presented. It features a low power 16-bitprocessor core supported by a number of hardware accelerators designed to perform complex operations required by advanced crypto algorithms. The microcontroller integrates an embedded Flash and an 8-channel 12-bit analog-to-digital converter making it a good solution for low-power sensor nodes. The article discusses the most important security topics in wireless sensor networks and presents the architecture of the proposed hardware solution. Furthermore, it gives details on the chip implementation, verification and hardware evaluation. Finally, the chip power dissipation and performance figures are estimated and analyzed.

## 1. Introduction

For many wireless sensor networks (WSN) in industrial, medical, and military applications, secure operation is mandatory [1]. The security requirements in WSNs are slightly different from the typical requirements of common communication networks. In all these networks, the security is characterized by data confidentiality, authenticity, integrity, and replay prevention [2]. Data confidentiality ensures that the data transmitted by a node do not leak to neighbouring networks. The standard approach to provide confidentiality is data encryption. Authentication is required to prevent malicious data injection by an adversary. It helps the receiver to confirm that the received data are sent from a trusted source. Authentication schemes are based on cryptographic signatures. Data integrity ensures that the data are not altered during their transmission. Integrity can be provided by implementing secure hash functions. Replay prevention must ensure that a transmitted message cannot be reused to bypass security schemes. The particular properties of wireless sensor networks, e.g., network size, harsh environment, dense deployment, changing topology as well as limited resources, make the network security easy to compromise if appropriate countermeasures are not applied.

**Figure 1:**
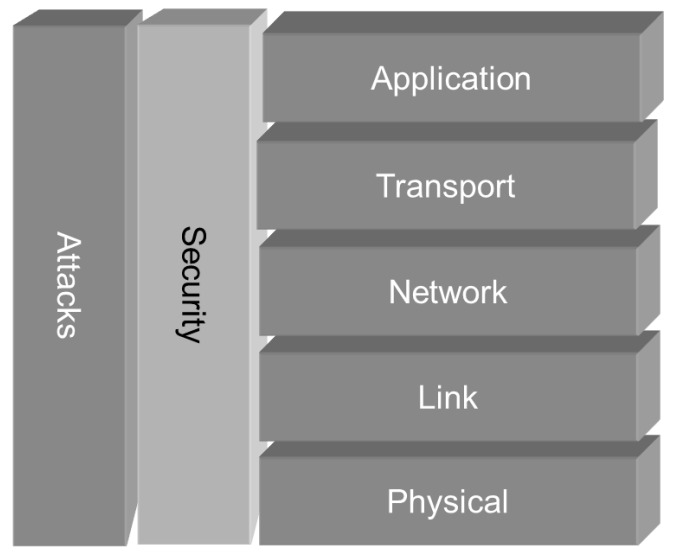
Attacks will try to compromise the security on all communication layers.

Operating in a hostile environment, sensor nodes are vulnerable to different types of malicious attacks. Eavesdropping, impersonation, injection of faulty information as well as denial of service are only some examples of hostile activities. A wireless sensor network attacker will try to compromise the network security and authentication, availability of data and the network service integrity [3]. Attacks can be launched on all layers of a sensor network protocol stack [4]. A simplified communication model is illustrated in Figure 1.

Attacks on the physical layer try to disable network functionality by radio frequency jamming or try to extract sensitive information by node tampering. Jamming attacks launched by a powerful radio source are very hard to defend [5]. Some techniques such as spread spectrum communication using frequency hopping can improve the network resilience [6], but these techniques require substantial computational resources, which are not available in typical wireless sensor nodes. Anti-tampering techniques attempt to prevent the disclosure of confidential data if a node is physically attacked [7,8]. The intruder may also try to install malicious software on the physically accessed node [9]. An effective anti-tampering can be implemented during chip design stage or by a tamper-proof package.

The standard cryptographic algorithms usually persist to exhaustive attacks but their hardware and software implementations have exhibited vulnerabilities to side-channel attacks, e.g., power analysis and fault injection attacks. Countermeasures against fault injection attacks on cryptographic devices have been described by Barenghi *et al.* in [10]. In [11], Karri *et al.* described the application of the fault-based side-channel cryptanalysis-tolerant Rijndael symmetric block cipher architecture at different levels of granularity, *i.e.*, checking against the inverse operation at the operation, round or full cipher level. This approach exploits the inverse relationship that exists between Rijndael encryption and decryption at various levels and develops concurrent error detection architectures that explore the trade-off between area overhead, performance penalty and error detection latency. The first passive and active combined attacks were reported in [12]. Amiel *et al.* showed that it is possible to attack an RSA exponentiation that was protected against power analysis attacks using a balanced algorithm with message randomization, by inducing *ad hoc* faults. A good way to protect against these attacks is to detect the injected fault immediately, rather than wait until the computation of the signature is completed, and upon detection, abort the process and thus avoid generating the power profile that discloses the secret key.

The attacks on the link layer can purposely introduce data collisions. By these collisions bits are flipped in the collided packets, which will cause a rejection during packet reception. The repeated collisions can lead to resource exhaustion and unfairness [13]. A typical solution for handling packet collisions is to use error-correcting-codes [14]. However, an attacker can theoretically introduce more errors as compensated by the code. The exhaustion attempts can be somewhat prevented by applying rate limits in the Media Access Control (MAC) protocol or by using time-division multiplexing. A further approach to prevent an unfairness threat is the usage of shorter frames so that an individual node can capture the channel for a short time only.

The network and routing layer is also prone to different types of malicious attacks [15]. Straight attacks on the routing layer are data spoofing, altering and replaying. Those attacks may disrupt the network traffic by creating routing loops, generating false error messages, extending and shortening routing paths, changing end-to-end latency, *etc.* An efficient countermeasure against data spoofing and altering is the introduction of a message authentication code. In addition, an adversary may attempt to create a sinkhole or a wormhole in the network topology by introducing malicious nodes. Sinkhole attacks use malicious nodes, which look more attractive to the neighbouring nodes according to the routing algorithm. Thus, the data traffic is forwarded towards a metaphorical sinkhole with an adversary in its centre. The wormhole attack uses two malicious nodes that create a low-latency link to a base station. Nodes located multiple hops away from the base station are convinced to forward their data to the created wormhole and, therefore, the data from distant nodes is tunnelled towards the wormhole created by an adversary. The wormhole attack is usually combined with the Sybil attack [16], where the compromised node provides multiple identities to the network or with the selective forwarding attack, where a malicious node refuses to forward messages and drop them. Hello flooding is an attack that targets the sensor networks, which use the broadcast of HELLO packets for announcing the node presence in the network [17]. By sending a HELLO packet from a powerful laptop-class sender to all nodes in the network, the attacker can convince some nodes in the network that they are in the range of the adversary node and make them to send their data into oblivion. An acknowledgment spoofing attack targets the protocols that rely on sending link layer acknowledgments to confirm the quality of a link. Spoofing the acknowledgment information may help an attacker to manipulate the network by presenting weak links as strong or dead nodes as alive. The resilience against the routing and network layer attacks can be improved by authentication, identity verification, link-layer encryption, authenticated broadcast and bidirectional link verification. However, the sinkhole and the wormhole attacks are sometimes difficult to defend. They may require a careful design of routing protocols. Protocols can potentially improve the resilience against wormhole attacks using geographic information [18].

Flooding and desynchronization are two possible attacks at the transport layer. The flooding attack uses a malicious sender, which sends a large number of connection requests to the victim. The victim allocates resources to maintain a state of the connection. The attack results in the exhaustion of memory resources of the victim node. Limiting the number of available connections or using the form of a puzzle solving algorithm [19] may help to defend flooding attack. Desynchronization refers to the attempts to disrupt an existing connection between nodes. As a consequence, the end node will repeatedly request for data retransmission. A possible solution is to require an authentication of the packets communicated among hosts.

Possible attacks on the application layer attempt to overwhelm the network with a large number of sensor stimuli [20] or to inject spurious or replayed packets into the network at leaf nodes [21]. The goal of these attacks is to provoke increased traffic and starve network resources. Again, data authentication can protect the network from those attacks. Table 1 presents the security attacks and countermeasures in WSNs at different communication layers.

There are numerous solutions trying to fight the security issues in wireless sensor networks. Software-based solutions try to establish new or try to optimize existing security algorithms for the applications in WSNs. However, software solutions have often to deal with insufficient processing resources and lack of memory [22,23,24]. Hardware-based solutions rely on the application of dedicated hardware accelerators, which enable fast execution of complex crypto-operations. Although additional hardware increases the chip area and power dissipation, it has been shown that hardware accelerators for crypto-operations (crypto-processors) consume less energy per bit than the standard software-based implementations [25,26,27]. However, the additional chip logic increases the microcontroller’s chip area and static power dissipation during sleep mode. The latter can be reduced by employing the power-gating [28].

This paper introduces a new hardware solution which implements advanced crypto-algorithms in a sensor node microcontroller at a low energy cost. It combines dedicated hardware crypto-accelerators and a low power 16-bit processor core, which asynchronously communicate with peripheral components. The next sections discuss and justify (comparing to the exiting solutions) the proposed solution (Section 2), present the architecture of the node microcontroller (Section 3), and describe the implementation and verification details (Section 4). Finally, the conclusions are drawn in Section 5.

## 2. Hardware Accelerated Crypto-Algorithms

Most security attacks can be prevented with cryptography-based security measures. There are two commonly used cryptosystems: symmetric (shared-key) and asymmetric (public-key) cryptography. Symmetric crypto-algorithms employ a shared secret key. Two communicating parties use the same key to encrypt and decrypt the data. Compared to public key cryptography, symmetric cryptographic algorithms are faster and computationally less demanding, which makes them the preferable solution in wireless sensor networks. In combination with cryptographic hash functions, symmetric algorithms can be used to generate “fingerprints”, which can guarantee data integrity. However, the problem of the shared key deployment restricts the usage of symmetric crypto algorithms in wireless sensor networks since no central distribution site exists. Asymmetric crypto algorithms use a pair of mathematically-related keys for encryption and decryption. Internet security protocol suites as Internet Protocol Security (IPsec) or Secure Sockets Layer (SSL) employ key exchange protocols as Internet Key Exchange (IKE) or Kerberized Internet Negotiations of Keys (KINK). Furthermore, a public key infrastructure (PKI) standard as X.509 can be used for authentication and shared secret key derivation. These keys are used in fast symmetric and hashing algorithms to increase the confidentiality, integrity and authentication of data. Both key exchange protocols and PKI are based on asymmetric cryptosystems. Asymmetric crypto algorithms are computationally expensive and usually seen as inappropriate for application in wireless sensor networks.

The development of a lightweight crypto algorithm like the PRESENT block cipher [29] that offers a level of security commensurate with a 64-bit block size and an 80-bit key has brought both security and hardware efficiency similar to many compact stream ciphers. Another promising solution is a high-security elliptic-curve-Diffie-Hellman function achieving record-setting speeds with several side benefits like free key compression, free key validation, and state-of-the-art timing-attack protection [30]. The lightweight crypto is basically related to software implementation of crypto algorithms on existing sensor node platforms. Those algorithms are in essence simplified versions of encryption standards. The lightweight crypto is designed to exploit limited resources of existing sensor nodes since the full implementation is not feasible as long as power hungry 32-bit microcontrollers are not applied. However, the performance and security level offered by such implementations is inferior to a full implementation of state-of-the-art cryptographic standards.

### 2.1. Symmetric Cryptography

The limitations of sensor nodes’ computational and power resources make shared-key cryptography a preferable solution for WSN applications. One of the first published security protocols for WSN was SPINS [31]. It is based on symmetric key cryptography and consists of two basic protocols, μTESLA, used for broadcast authentication and SNEP providing two-party data authentication, data confidentiality, integrity and evidence of data freshness. SNEP uses the counter mode (CTR) of a symmetric block cipher to provide data confidentiality. TinySec proposed in [22] is another link layer protocol for WSNs, but it uses the weak RC5 algorithm for data encryption. The Skipjack primitives are used to provide authenticated encryption and authentication. A general-purpose security protocol designed for the Telos platform is MiniSec [23]. MiniSec employs the Offset Codebook (OCB) encryption mechanism and uses Skipjack as the underlying block cipher. The popular Zigbee standard provides security on different protocol layers [32]. It proposes the use of two session keys: a link key for node-to-node communication and a network key for broadcast messages. Furthermore, an initial master key is desirable. This key enables generation of the two session keys. Zigbee also proposes a centralized trusted site, which would be responsible for session key generation and admission of new nodes that want to join the network. The proposed block cipher in Zigbee protocol is a symmetric advanced encryption standard (AES) of a key length of 128 bits.

The major obstacle in applying symmetric cryptography in wireless sensor networks is the key management and the key deployment. Due to the network dynamic nature, the random behaviour of communication links and the hostile environment traditional approaches based on a centralized trusted authority are seen as inappropriate. A possible, simple solution of this problem may be a distribution of the shared secret keys before the node’s deployment, but this technique is very inflexible and not suitable in dynamic application scenarios. Therefore, more flexible techniques for a key deployment in WSN are discussed in the following section.

Establishing a secure link between two nodes requires agreement on a secret key by both communication parties. A traditional Internet-style approach based on a trusted centralized site is not suitable for WSNs. The unknown network topology, range limitations, intermittent sensor-node operations and network dynamics make network integration very difficult. At the other hand, public-key schemes are seen as too computationally expensive to be efficiently implemented in wireless sensor networks. The limited computation and energy resources constrain their efficient implementation. Therefore, key pre-distribution has been seen as the most practical solution for the key agreement problem. A simple solution is a global key stored in each node, but this is not particularly efficient because of the whole network is compromised if only a single node is compromised. A pair-wise key sharing between every two nodes is impractical since requires a pre-distribution and storage of *n*-1 keys on each of the *n* sensor nodes. The number of keys will quickly exhaust the nodes’ memory limits. Moreover, key-pairs could be used between the neighbouring nodes only, so joining of new nodes becomes impossible.

A scheme based on probabilistic key distribution is presented in [33]. Here, each node receives a random subset of keys from a large pool. For establishing a communication link, nodes look for a common key out of their subset and use the selected key as a shared secret key. Consequently, the establishment of a secure communication is not certain but guaranteed with some probability. An improved and generalized q-composite random key pre-distribution scheme is presented in [34]. It improves the network’s resilience to small-scale attacks at the cost of greater susceptibility to large-scale attacks. A pair-wise key pre-distribution scheme presented in [35] improves network resilience at the cost of additional computational requirements. LEAP proposed in [24] is a key management protocol designed to support multiple keying mechanisms and in-network processing. It uses RC5 block cipher to ensure the data encryption and encrypted authentication.

The work presented in [36] proposes a security framework based on a triple-key management scheme developed for cluster-based network topologies. The scheme consists of three keys: two pre-deployed keys in all nodes and an in-network generated cluster key for a cluster to address the hierarchical nature of the network. The framework also defines the routing and localization algorithms and a mechanism for malicious node detection. The presented approach promises low packet overhead and low transmission latency. In [37], the authors propose a key management scheme based on dynamic key reconfiguration where already deployed nodes refresh their keys prior to another deployment phase. A scalable key management scheme that uses the unital design theory is presented in [38]. The presented algorithm relies on the mapping of basic unitals to the key pre-distribution scheme providing high network scalability and secure connectivity.

### 2.2. Public Key Cryptography

Public-key algorithms are often seen as inappropriate for implemention on wireless sensor nodes. This is mainly driven by the large code size and expensive computation requirements of these implementations. Standard public-key schemes such as the Diffie-Hellman key agreement protocol [39] or the Rivest, Shamir und Adleman (RSA) signature algorithms [40] require a large number of multiplications to perform a single cryptographic operation. The elliptic curve cryptography (ECC) as an advanced replacement of RSA offers smaller keys and faster computation. Furthermore, it promises savings in data bandwidth, memory utilization and energy consumption. The performance superiority of ECC over RSA when implemented on an 8-bit ATmega128L processor is presented in [41]. The authors of [42] have shown that a challenge-response authentication based on ECC-160 uses only 1/12 of the energy of its RSA-1024 counterpart.

Although the software implementation of public key cryptography on small scale sensor devices is proved to be possible, the introduced power overhead is still too high. A promising solution to make asymmetric cryptography suitable for small scale platforms is the usage of hardware accelerators. It can improve efficiency and performance of crypto operations in order of magnitudes on those nodes. A hardware platform containing an additional off-the-shelf coprocessor from ATMEL is presented in [43]. The coprocessor is used to improve RSA computation. A VHDL implementation of the Rabin’s scheme and the NtruEncrypt algorithm is presented in [44] and a design of ECC-163 coprocessor is presented in [25]. A comparison between the ECC software solution on a TI MSP430 microcontroller and an FPGA implementation is given in [27]. It shows that the power consumption can be improved three orders of magnitudes when ECC is implemented on an FPGA.

### 2.3. Combining Crypto-Algorithms

Although proven as feasible, software implementations of complex cryptographic functions on small-scale sensor nodes are constrained by the limited computational and energy resources. Symmetric cryptography is faster and more power-efficient than public-key schemes, but suffers from the key agreement issue. The proposed key pre-distribution schemes may improve the network resilience, but the network security can still be compromised. The public-key cryptography could potentially solve the problem of key management, but it is too expensive to be efficiently implemented in combination with symmetric crypto algorithms on a typical sensor node platform. The sensor nodes based on powerful 32-bit microprocessors have enough resources to process complex crypto operations but their energy efficiency is quite low. The dedicated hardware-based solutions promise good energy efficiency and superior performance at the cost of area overhead and reduced flexibility. Furthermore, to design an embedded low-power sensor node microcontroller that can efficiently deal with the security issues in WSNs is a challenging task. Nevertheless, for many sensor network applications where the security is a key, a custom-based hardware may be the best trade-off between low power operation and required functionality.

We propose the solution which is a tailor-made sensor node microcontroller that provides the computational infrastructure for an efficient combination and implementation of advanced security algorithms based on symmetric and asymmetric cryptography. The proposed hybrid approach enables safe distribution of a shared key between sensor nodes using public key cryptography. Once the key has been distributed to every node in a network, a more power-efficient shared key crypto algorithm is used for data encryption. To the best of our knowledge, our chip is the first embedded sensor node microcontroller that combines a general-purpose microcontroller with hardware support for both public- and shared-key cryptography on a single chip. This approach provides a higher level of security along with reduced power dissipation comparing to the systems implementing symmetric-only ciphers suffering from the key agreement problem or those based on power-hungry 32-bit processors. Furthermore, the developed system-on-chip is the first embedded sensor node microcontroller with embedded both symmetric and asymmetric crypto (hardware) accelerators successfully evaluated on a dedicated sensor node platform.

### 2.4. Contributions

The main contribution of this work is providing an embedded low power microcontroller to be used in sensor network applications with high security demands. The designed 16-bit microcontroller, compatible with the TI MSP430x family, is capable of power efficient execution of complex cryptographic algorithms with superior performance. The microcontroller implements hardware acceleration for well-established crypto algorithms (ECC, AES and SHA-1) as well as a communication accelerator improving connectivity in noisy environments. The hardware implementation of symmetric and asymmetric cryptography provides several orders of magnitude improvement in power efficiency over compatible software implementations on commonly used sensor node platforms based on the same type of microcontroller. The performance, when measured in execution time, is also improved dramatically in favour of the proposed hardware implementation. Additionally, the security level provided by a full scale hardware implementation of the cryptographic features overcomes the security level provided by a lightweight software implementation of crypto algorithms, which performance is fairly limited due to the lack of hardware resources in the node.

## 3. Microcontroller Design

Design of a sensor node microcontroller (called TNODE after the node’s first version developed in the Tandem project) is based on the IPMS430 processor core, which is fully compatible with the Texas Instruments MSP430 processor architecture [45]. The IPMS430 is an asynchronous processor addressing up to 1 MB of memory space. The processor communicates with peripherals via a dedicated peripheral interface. Peripherals are designed as standard synchronous devices having implicit clock synchronisation on their interfaces to the processor. A dedicated clock provided to each of the peripherals is only generated when a peripheral is accessed by the processor. The global clock runs independently and it does not interfere with the generated clocks. The peripheral interrupts are chained with predefined priorities.

### 3.1. Related Work

Over the time, several versions of TNODE design based on the same core architecture were implemented. The very first version (TNODE1) was a general purpose microcontroller enhanced by a communication accelerator (not designed for security applications) [46]. The second version (TNODE2) was the first that featured hardware crypto accelerators [26]. This design used an external flash memory accessed through a parallel data interface for its program memory. It introduced a large number of pads and significant area overhead and, consequently, increased the power dissipation. TNODE2 was fabricated in IHP’s 250 nm BiCMOS technology. The third version (TNODE3) was implemented and fabricated in IHP’s 130 nm BiCMOS technology [47]. This design relies on an external program memory as well, but contains four crypto accelerators and a communication accelerator to support the link layer operations defined by IEEE 802.15.4 standard. The recently developed fourth version (TNODE4) was the first with an internal non-volatile memory and an ADC convertor [48]. This design was fabricated in IHP’s 250 nm BiCMOS technology and reaches the maximum frequency of 11.4 MHz. Our latest design (TNODE5) integrates the ECC crypto core optimized for better robustness against side channel attacks and a hardware accelerator ensuring reliable communication in noisy environments. The design also implements an additional 32-bit timer, which allows control of long sleep periods typical for WSN applications. The TNODE5 has a reduced number of pads (64) and an on-chip digitally controlled oscillator (DCO), which provide additional power savings and higher flexibility to application demands. In the following sections, the architecture, design, and implementation of TNODE5 are only discussed.

### 3.2. Design Architecture

**Figure 2:**
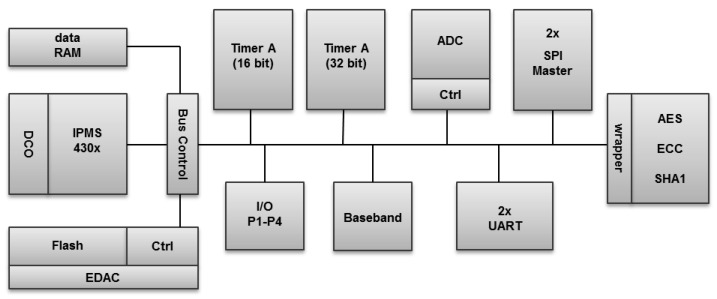
Architecture of TNODE5 microcontroller.

TNODE5 includes the on-chip peripherals such as standard serial interfaces (UART and SPI), a 16-bit and a 32-bit timer, digital IO ports, communication and crypto accelerators as well as controllers for the integrated ADC and Flash memory. The chip was designed to fit into a 64-pin package, where all the peripheral pins, except ADC ports, are shared with the general-purpose IO pins. A smaller footprint of the chip provides better integration capabilities and helps reducing the overall size of an assembled sensor node. The architecture of TNODE5 is shown in Figure 2.

### 3.3. Standard System Components

All system components connect to the processor over a memory mapped bus structure. The processor is the master device and initiates all transactions on the bus. Multiple masters are not allowed. All peripherals act as slave devices and may signal interrupt requests to the processor. The interrupts are handled by the processor in a prioritized order. The interrupt handling and the address multiplexing are maintained by the Bus Control block.

### 3.4. Digital IO Ports

The system implements four 8-bit digital IO ports. The ports P1-P4 are fully compatible with the Texas Instruments (TI) MSP430x implementation of digital IO. The IO port signals of the chip are shared with peripheral signals as the serial interface and timer signals. The selection of each signal can be individually programmed.

### 3.5. Timers

TNODE5 features two timers, a 16-bit timer compatible to the *Timer A* specification of TI and its extended version, a 32-bit timer. The 32-bit timer has the same functionality as its 16-bit counterpart but it can provide much longer counter intervals. This feature becomes very useful in WSN applications with extremely low duty cycles.

### 3.6. Serial Interfaces

The serial interfaces of TNODE5 provide connectivity to digital sensors and external devices such as radio transceivers. The chip provides two UART and two SPI ports.

### 3.7. Memory

The TNODE5 processor is based on 16-bit von-Neumann architecture. It has a shared address space containing the data and program memories. The extended address bus architecture allows access to 1 MB of memory space. The chip integrates 64 kB of the Flash-based program memory and 16 kB of the random access data memory. The integrated sector-erasable Flash is organized in 16 k × 44-bit data words. The sector size is variably selectable from 2^0^ to 2^9^ kbits. The Flash controller implements an error detection and correction (EDAC) algorithm that operates on additional 12 bits of each data word. The implemented EDAC can correct 1-bit errors and detect 2-bit errors, which significantly improves the system reliability. The initial programming of the Flash memory must be performed via I^2^C debug interface of the TNODE5 processor. During the programming operation via the I^2^C debug port the Flash controller is set into the debug mode in which each timing-related function is stalled until the erase/write procedure is finished. Alternatively, the Flash memory can be programmed by the processor core by using the register interfaces of the controller. The programming can be done on a sector-by-sector basis, which is very usefully for an on-the-fly update of software modules.

### 3.8. Crypto Components

The TNODE5 microcontroller is equipped with hardware support for AES, ECC, and SHA-1 crypto operations. Each crypto accelerator has a 32-bit interface and can be separately enabled or disabled. The crypto cores build a single logic block that communicates to CPU over a 32-to-16-bit interface.

### 3.9. AES

The Advanced Encryption Standard (AES) was standardized in 2001 and it supersedes the insecure DES-Algorithm. It is a symmetric block cipher, based on the Rijndael algorithm [49]. The AES operates on a fixed block size of 128 bits and supports three different key lengths 128, 192, and 256 bits. Our AES implementation supports a key size of 128-bit only. The encryption as well as the decryption of single 128-bit block takes 66 clock cycles only. For comparison, a software implementation of AES on TI MSP430F1611 microcontroller takes 6600 cycles for encryption and 8400 cycles for decryption [50]. That makes a speed up of two orders of magnitude in favour of our hardware implementation. The detailed overview of the implemented AES core and its advantages over a software solution is given in [51].

Although still considered as a safe encryption standard, AES-128 is expected to become vulnerable in the near future. Therefore, the more secure AES-256 standard is to be considered as a standard option for the future releases of the TNODE chip. Our preliminary investigation on the AES-256 implementation using the same architecture shows that the encryption and decryption operations would require 90 clock cycles each to complete. Accordingly, the chip area and power consumption will increase due to the increase in the size and the number of registers required for storing the key and the data.

### 3.10. ECC

Elliptic Curve Cryptography (ECC) is an asymmetric cryptosystem that exploits the complexity of algebraic operations on elliptic curves over finite fields. The calculation difficulty of finding the discrete logarithm of a random elliptic curve element makes ECC security very high even with relatively short keys. The ECC core of our chip operates on the 233-bit binary field and is inspired by the work presented in [25,52]. The operation of elliptic curve point multiplication (ECPM) is implemented in accordance to the Lopez-Dahab algorithm [53]. The multiplier itself is an Iterative Karatsuba multiplier [54]. For each 233-bit operation the multiplier takes nine clock cycles. The core is optimized for low power but limited to the 233-bit binary field.

The integrated ECC design is an implementation of the Montgomery kP-algorithm that performs bitwise processing of each secret key bit. The key bits are processed with the same type, amount and sequence of operations, independently of the bit’s value. For this reason, each bit processing takes same amount of the time and requires a similar amount of power consumption. Thus, the core is protected against side channel attacks based on simple power analysis (SPA). However, a complex differential power analysis (DPA) attack could potentially crack the encryption. The vulnerability of the implemented ECC to DPA attacks as well as the countermeasures is thoroughly discussed in [55]. Other possible ways of increasing the resistance of our implementation against DPA attacks could be by randomizing its processed data. For example, randomizing the private key, randomizing the projective coordinates of the elliptic curve points or blinding the elliptic curve point can improve the robustness of the ECC [56]. Implementing such countermeasures implies additional costs regarding power consumption and area.

Several ECC hardware-based solutions have been proposed in literature [57]. In [58], the authors proposed a low cost ECC processor to be used in sensor node systems. This solution had a small area footprint, but relatively weak performance. It requires 57,720 clock cycles for an ECPM over a 133-bit binary field and 95,159 clock cycles for an ECPM over a 163-bit binary field. For comparison, our solution takes 13,164 clock cycles for an ECPM over a 233-bit binary field. In [59], the authors compare three alternative ECC-163 implementations for MSP430-based sensor nodes: (1) a software solution; (2) a dedicated hardware; and (3) a drop-in module solution. The drop-in module, based on a modification of the MSP430 processor, is introduced to compromise between the software solution and relatively costly dedicated hardware. The comparison of performance and power figures (estimated after the synthesis) of the proposed ECC implementations is given in Table 2.

### 3.11. SHA-1

Secure Hash Algorithm (SHA-1) is probably the most widely used algorithm for secure hash calculation. SHA-1 generates a 160-bit message digest of a 512-bit input block. It is based on principles similar to those of MD4 and MD5, but has a more conservative design. The SHA-1 core of the TNODE5 microcontroller requires 160 clock cycles to generate a 160-bit hash message. It can be used to ensure data integrity and (in combination with ECC) to generate digital signatures executing the elliptic curve digital signature algorithm (ECDSA). In addition, the SHA-1 has a programmable initial value register, which makes it suitable for generation of pseudo random numbers. These numbers are required by various cryptographic operations.

**Figure 3:**
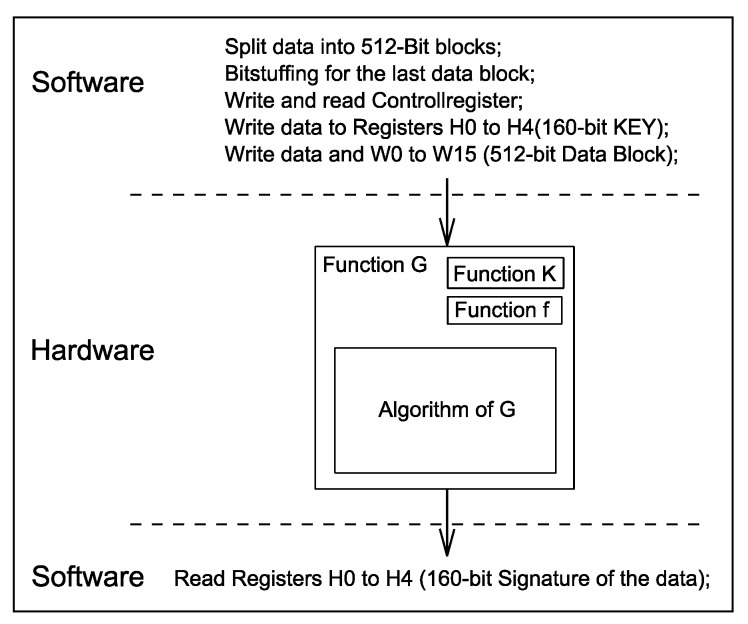
Hash generation flow.

The internal architecture of the implemented SHA-1 core is designed to process 512-bit input data blocks. The bit-stuffing technique is employed to align the data size to the 512-bit boundary. The bit-stuffing operation is performed in software. The information on the actual data size is stored in the control register. The 32-bit registers H0-H5 are used to store the 160-bit secret key. The 32-bit registers W0-W15 are loaded with a 512-bit input data block. The hardware blocks within the SHA-1 core are responsible for generating the signature by performing the algorithm that is described with functions f, K und G. The functions f and K generate intermediate results of the hash algorithm. The function G performs the major hash generation operation and saves the signature back to the H register. This is illustrated in Figure 3. The generation of a signature for a 512-bit block takes 80 clock cycles.

The SHA-1 algorithm was the standard cryptographic hash function during the development of the TNODE microcontroller. The recent reports show that the SHA-1 can be broken with approximately 2^57^ equivalent SHA-1 evaluations [60]. The more powerful SHA-3 algorithm was standardized in 2014 and it is becoming the next leading secure hash function. For a new version of the TNODE the implementation of SHA-3 will be considered as the standard option. However, the hardware implementation of SHA-3 would contribute to additional costs regarding performance, power and area of the final chip.

### 3.12. Communication Accelerator

To support an efficient and secure communication, the TNODE5 design implements a baseband controller that complies with the DIN EN 13757-4 standard. The DIN EN 13757-4 is a communication standard for meters and remote reading of meters. It is specified for short range devices running at frequencies of 868–870 MHz with data rates ranging from 2.4 to 66.6 Kbit/s. The baseband supports 3-out-of-6 and Manchester coding defined by the standard. The baseband is extended to support the direct sequence spread spectrum (DSSS) to improve communication in a highly jammed environment (Barker 7 and 11 modulation). The DSSS uses the spreading code for converting the narrow band information into wideband information. In DSSS each of the information bit is XOR-ed with the barker code at the point of the transmission. As a result, the whole information is converted into a very wide band and the information signal remains the same. This spread spectrum is able to reject interferences or jamming attacks and allows a recovery of signal where less than fifty percent of the data bits have been damaged during transmission. Besides its high robustness it allows a data transmission in a very noisy environment, which makes a detection of the data transmission very hard. The implemented baseband core includes clock gating and wake-up support for maximum power efficiency. It can process an input data stream with a clock offset higher that 2%. The offset is compensated during the frame reception. This feature can improve communication with devices that have large clock drift caused by any reason. The core implements a programmable clock prescaler that allows the programming of various bitrates derived for different input clock frequencies.

### 3.13. Analog Components

The TNODE5 design includes two analog components, a 12-bit analog-to-digital converter and a digitally-controlled oscillator. Both components contain mostly MOS and passive components. The bipolar transistors are used in bandgap reference circuits only.

### 3.14. ADC

Connectivity to external analog sensor sources is provided by an integrated 12-bit analog-to-digital convertor (ADC). The implemented device is a fully differential 100 kSps, 12-bit, successive approximation register ADC with self-calibration capabilities. The sampling rate goes up to 1 MSps at the nominal supply voltage of 2.5 V. The operation is also possible at a supply voltage of 2 V but both generate results with reduced accuracy. The size of the ADC core is 1.12 mm × 1.1 mm.

### 3.15. DCO

The TNODE5 design integrates a digitally controlled oscillator (DCO). It supports 64 different frequencies ranging from 5.8 MHz up to 13.9 MHz. The DCO has a size of 0.024 mm^2^ and consumes in active mode around 265 µW. When disabled, its standby power is around 2.5 µW. The oscillator is programmable via a 64-bit 1-hot decoder that improves DCO frequency stability. DCO generates clock signals with a duty cycle of nearly 50% and has a settling time of 15 µs. More details on the implemented DCO are given in [61].

## 4. Implementation and Verification

The TNODE5 chip is implemented using the standard design flow of the IHP’s 250 nm BiCMOS technology. The logic synthesis is performed with Synopsys Design Compiler for a target frequency of 20 MHz. The total cell area of the synthesized chip excluding IO pads is estimated to 13.33 mm^2^. Table 3 presents synthesis results for the cell area of different TNODE5 components. The results show that the analog and memory components (SRAM, Flash, DCO, and ADC) consume 71.8% of the total cell area. The remaining 38.2% is taken by the logic components. The crypto cores take 15.6% and the baseband core takes around 3.9% of the total cell area. This results in an area overhead due to the crypto and communication cores of nearly 20%.

**Figure 4:**
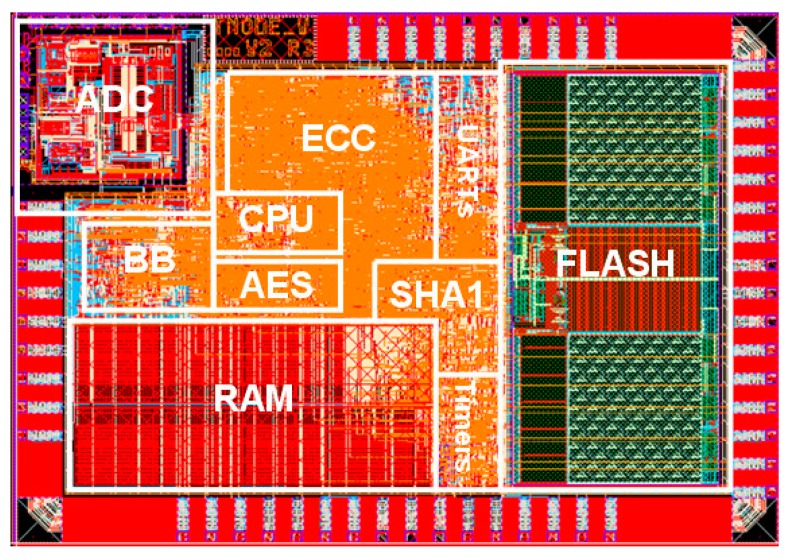
Layout of the TNODE5 chip.

The layout of the chip is designed with the Cadence SoC Encounter tool. The size of the final chip is 23.96 mm^2^, which is twice the size of the estimated cell area. The difference between the estimated and the final chip size is mainly reasoned by the additional area required by IO pads, the power supply network and the routing constraints. The photo of the TNODE5 layout is given in Figure 4. The TNODE5 has only 64 IO pads (39 digital, 13 analog, and 12 power/ground), which makes it possible to package the chip in a small 64-pin quad-flat-no-leads package (QFN). Furthermore, a very small ball grid array (BGA) package can be used as well. Further miniaturizations are possible with a PCB direct bonding and the flip chip technology. While a direct bonding is possible with the current chip version the flip chip technology requires special design rule, which are planned to use in future versions. Any miniaturization makes the chip more robust against tampering and improves its usability in sensor node applications.

The verification process of the TNODE5 chip includes functional verification during the design process and the verification of the fabricated chips. The functional verification during the design stage includes the simulation of digital and analog parts and the simulation of the whole system. The simulation tests were designed to cover the functionality of the overall system and each peripheral in particular. The production tests on the fabricated chips had to confirm the functional correctness and the compliance to the specified electrical and system parameters, e.g., power consumption and maximal clock rate. The final evaluation of the chip has been done on a dedicated hardware evaluation platform.

### 4.1. Simulations

**Figure 5:**
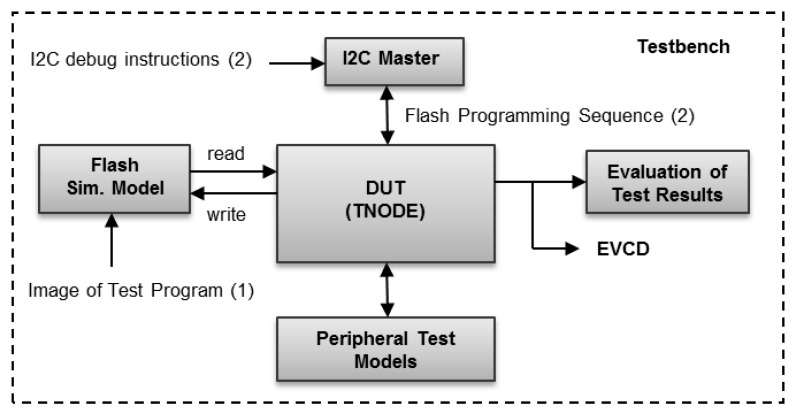
TNODE5 testbench: (**1**) Test execution from Flash; (**2**) Test execution via I^2^C debug port.

The TNODE5 simulations include a set of test programs which are executed from the internal Flash memory. Numerous tests were generated to verify the functionality of peripherals and the overall system. During simulation, the image of a test program is preloaded into the functional model of the Flash memory. Then the program is executed by the system. The test results are continuously being written to the external ports and evaluated by the testbench. For testing the Flash, a simulation model of an I^2^C debug device (I^2^C master) was created. The device is used to program the Flash memory. Furthermore, Flash programming sequences were generated and used to verify common Flash operations like erase, write, and read. The same procedure is used for testing the processor’s instruction set and generation of the Flash programming test patterns required by production tests. The proposed simulation approach is illustrated in Figure 5. The functionality of analog components is tested in analog simulations. In digital simulations, only the simple functions of ADC and DCO control logic were tested.

### 4.2. Production Tests

Testing of the fabricated TNODE5 chips was performed on the Agilent Verigy 9300 industrial tester. The Verigy 9300 is a cycle-based industrial tester for digital circuits, which supports both wafer and package testing. The tester operates on the data extracted from an EVCD (extended value change dump) file. The EVCD file is generated by the simulation framework and includes the value change information for all input and output signals. In a standard test procedure, the input stimuli extracted from the EVCD file are applied to a chip. The output signals of the chip are sampled by the tester and compared with the expected output values. Since the TNODE5 design cannot execute an external program, the stimuli data must be written into its Flash memory first. Therefore, a set of initial I^2^C program sequences was generated for storing the test program in the Flash memory. Such a programming sequence includes all I^2^C instructions to erase the Flash memory, to write the test program and to verify the written data. After loading the Flash memory, a functional test is started by booting the processor. The booted program performs the functional test and writes the results to the processor outputs, which are sampled and verified by the tester. The TNODE5 chips were tested on the complete set of production tests.

The analog components, the ADC and the DCO, cannot be comprehensively tested on the digital tester. Nevertheless, the AD conversion of predefined voltage levels feed at the ADC analog inputs can be tested by comparing the ADC output with the expected values obtained from analog simulations. More detailed tests of the ADC are performed on a special test board by using appropriate testing equipment. The same board was used for testing the DCO as well.

### 4.3. Power and Performance

The power dissipation of the TNODE5 design was estimated in the layout-phase and measured on the fabricated chip. The post-layout power estimation is performed with the Encounter Power System tool that is a part of the Cadence Encounter design toolset. The power estimation is done for typical operating conditions (T = 25 °C, V_core_ = 2.5 V, V_pad_ = 3.3 V) and for an operating frequency of 1 MHz. The dynamic power was estimated by using the activity data extracted from the post-layout simulations. Furthermore, the power dissipation was estimated for different crypto operations executed by the integrated crypto accelerators. In addition, the power dissipation is estimated during the data transmission and reception by the integrated baseband core. Finally, the power consumption during an SPI data exchange was estimated. During all tests the ADC and the Flash were active and had constant power consumption. The results of power estimation are summarized in Table 4.

The power estimation results show that the ADC and the Flash consume 13.6 mW, which is much more than the chip logic. The ADC supports a standby mode where its power consumption drops to several µW. The Flash component must be active as long as the processor is reading data from it. If it is not selected, the Flash component enters the standby mode automatically. As expected, the maximum power consumption in the chip logic was estimated for the ECC operations, which corresponds to the occupied cell area.

The early power estimation on the fabricated chips was performed during the production tests. The TNODE5 chip includes dedicated power supplies for the core (VDDCORE), pads (VDDPAD), and analog and mixed signal parts of ADC (VDDA and VDDM). The VDDCORE source supplies the standard logic cells, Flash, and digital parts of the ADC and pads. Therefore, it was not possible to measure the power dissipation of the ADC and Flash separately. Measurement results of the node’s average power dissipation for several production tests are given in Table 5. The power dissipation is estimated by measuring the current of each power source separately at the nominal supply voltage (VDDCORE = 2.5 V, VDDA = VDDM = 2.5 V, VDDPAD = 3.3 V). The operating frequency was fixed at 1 MHz. The results in Table 5 present the average power dissipation extracted from the measurements of 51 randomly selected chips. The results show a good correlation with the estimated power dissipation. The highest power dissipation of the VDDCORE source was measured during crypto tests. The power dissipated at pads is higher during the SPI and baseband tests, which correlates to the higher activity on external ports during these tests. In addition, the power of three randomly selected chips was measured at an operating frequency of 10 MHz. The results are summarized in Table 6.

The estimated power consumption of TNODE5 is fairly high when compared to commercially available state-of-the-art TI MSP430 processors that consume up to 1 mW @ 1 MHz. However, one has to consider that the TNODE5 was fabricated in a mature 250 nm technology process with limited access to low power cells, analog components and memory devices. If scaled to an advanced 130 nm low power process, the power of TNODE5 could be reduced at least 6-8 times. If fabricated in 90 nm, the active power consumption of TNODE5 would be expected to drop under 1 mW. The application of advanced low power techniques for leakage reduction could additionally improve the power efficiency of the microcontroller. Nevertheless, the energy efficiency of the current design when executing implemented crypto algorithms is still superior to equivalent software implementations on commercially available 16-bit microcontrollers.

## 5. Conclusions

To answer the security challenges in wireless sensor networks, the TNODE design, a sensor node microcontroller that combines a general-purpose processor with hardware support for both public-key and shared-key cryptography, was integrated on a single chip. The implemented cryptographic techniques provide a high level of security at a minimum cost of additional silicon area and increased static power dissipation. The TNODE5 chip was fabricated in IHP’s 250 nm BiCMOS technology and successfully tested. The measurements confirmed the outstanding performance of the TNODE design in comparison to the existing solutions based on software implementation of cryptographic standards. Future improvements of the proposed solution should include the design of more complex clock control logic and the implementation of power gating. Also, more robust encryption standards such as AES-256 and SHA-3 are to be implemented. Furthermore, the flip chip technology can be used to minimize the physical size of the sensor node microcontroller to increase its robustness against malicious tampering. Finally, the ECC is to be enhanced with the features preventing advanced side channel attacks.

## Figures and Tables

**Table 1:** Security attacks and countermeasures in wireless sensor networks.

Network Layer	Security Attack	Countermeasure
Physical	Jamming	Spread-spectrum, frequency hopping
	Tampering	Tamper-proof design
Link	Collisions	Error-correcting codes
	Exhaustion	Data rate limits, time division multiplexing
	Unfairness	Short frames
Network and Routing	Spoofing, altering, replaying	Authentication, link-layer encryption
	Sinkholes	Authentication, link-layer encryption
	Wormholes	Authentication, geographic routing, tight synchronisation
	Sybil	Authentication, public key cryptography
	Selective forwarding	Authentication, link-layer encryption, multipath routing
	HELLO attack	Authentication, bidirectional link and identity verification
	Acknowledge spoofing	Authentication
Transport	Flooding	Client puzzles, authenticated broadcast
	Desynchronization	Authentication
Application	Stimuli attack	Authentication
	Packet injection	Authentication

**Table 2:** Comparison of different ECC implementations.

	Architecture	Binary Field	Frequency (MHz)	Technology Node (nm)	Runtime (Cycles)	Power (µW)	Energy (µJ)
[58]	Dedicated Hardware	ECC-133	0.5	130	57,720	30	3.46
[58]	Dedicated Hardware	ECC-163	0.5	130	95,159	n.a.	n.a.
[59]	Software	ECC-163	1	130	7,216,905	49.1	354.35
[59]	Dedicated Hardware	ECC-163	1	130	54,376	181.7	9.9
[59]	Drop-in Module	ECC-163	1	130	182,130	70	12.8
This Work	Dedicated Hardware	ECC-233	20	250	13,164	6230	4.1

**Table 3:** Cell area of TNODE5 components estimated after synthesis.

Chip Component	Cell Area (mm^2^)	Percent (%)
Flash	5	37.6
RAM	3.3	24.8
ECC	1.45	10.9
ADC	1.23	9.2
Baseband	0.53	3.9
Uart1 + Uart2	0.4	3
SHA-1	0.37	2.8
CPU	0.3	2.3
AES	0.25	1.9
Flash Controller	0.12	0.9
Timer (32 bit)	0.11	0.8
Timer (16 bit)	0.075	0.6
SPI1 + SPI2	0.048	0.4
P1 + P2	0.048	0.4
P3 + P4	0.022	0.2
DCO	0.024	0.2
Bus Control	0.018	0.1
**TNODE5**	**13.33**	**100**

**Table 4:** Post-layout power estimation of TNODE5 microcontroller.

Operation	Logic (mW)	ADC (mW)	Flash (mW)	Total (mW)
SHA-1 Calculation	0.98	6	7.6	14.58
AES Decryption	1.18	6	7.6	14.78
AES Encryption	1.25	6	7.6	14.85
ECC Point Multiplication	2.68	6	7.6	16.28
ECC First Point Inversion	2.11	6	7.6	15.71
ECC Second Point Inversion	2.06	6	7.6	15.66
Transmit Mode	0.69	6	7.6	14.29
Receive Mode	0.71	6	7.6	14.31
SPI	0.98	6	7.6	14.58

**Table 5:** Post-production power measurements of TNODE5 chip at a frequency of 1 MHz.

Test @ 1 MHz	P_VDDCORE_ (mW)	P_VDDPAD_ (mW)	P_VDDA_ (mW)	P_VDDM_ (mW)	P_TOTAL_ (mW)
Rx	7.5954	3.3426	1.7194	2.2422	14.8996
Tx	7.2630	3.0922	1.7236	2.2540	14.3328
Crypto	7.6204	2.8366	1.7292	2.2747	14.4609
SPI	6.0176	3.2306	1.7163	2.2625	13.2270

**Table 6:** Post-production power measurements of TNODE5 chip at a frequency of 10 MHz.

Test @ 10 MHz	P_VDDCORE_ (mW)	P_VDDPAD_ (mW)	P_VDDA_ (mW)	P_VDDM_ (mW)	P_TOTAL_ (mW)
Rx	13.5437	3.3492	1.0595	2.2430	20.1954
Tx	13.8279	3.2426	1.0635	2.2525	20.3865
Crypto	26.582	3.3108	1.0602	2.1442	33.0972
SPI	14.0432	4.6625	1.0572	2.2842	22.0471
